# Bluetongue virus seroprevalence and risk factor analysis in cattle and water buffalo in southern Italy (Campania region)

**DOI:** 10.1007/s11259-023-10215-w

**Published:** 2023-09-08

**Authors:** Gianmarco Ferrara, Elvira Improda, Federica Piscopo, Riccardo Esposito, Giuseppe Iovane, Ugo Pagnini, Serena Montagnaro

**Affiliations:** Department of Veterinary Medicine and Animal Productions, University of Naples Federico II, Via Federico Delpino N.1, 80137 Naples, Italy

**Keywords:** Bluetongue, Co-exposure, Water Buffalo, Ruminants, Italy, Orbivirus, Arbovirus, Bunyavirus, Schmallenberg virus

## Abstract

Bluetongue is an arthropod-borne viral infection that is notifiable in several countries and causes significant economic losses and major concerns for ruminant trade. In this study, we investigated bluetongue 1seroprevalence in the Campania region, southern Italy, in cattle and buffalo populations, and assessed which factors were correlated with a high risk of exposure. The infection was widespread, as evidenced by the high individual (43.6%) and herd prevalence (85.4%). The highest prevalence was found in adult animals. Among the climatic factors analyzed, average temperature played a prominent role, being capable of affecting the probability of being positive for this infection. Surprisingly, exposure to Schmallenberg virus did not predispose animals to be positive for bluetongue virus, even though these infections share the same vector (*Culicoides*). Our data, consistent with those in the literature, suggest the transversal spread of bluetongue virus in the Mediterranean area, and indicate a limited co-exposure rate between Bluetongue and Schmallenberg viruses.

## Introduction

Arboviruses pose a threat to both human and animal health, causing infections that have spread rapidly in recent decades, reaching areas where they were previously considered tropical (Socha et al. 2022). The name of this virus group, which has no taxonomic meaning, derives from the common transmission of its members (arthropod-borne), and consequently, the spread of these viruses depends on the distribution of vectors (Koenraadt et al. 2014). This group includes viruses responsible for concerning zoonoses (such as dengue, yellow fever, or zika), as well as several viruses of veterinary importance (such as Schmallenberg and bluetongue) (Socha et al. 2022). Bluetongue virus (BTV) is a non-contagious RNA virus spread by the bite of *Culicoides*, that has significantly invaded Europe in recent decades (Mignotte et al. 2021).

BTV was responsible for well-known outbreaks in the late 1990s and early 2000s and is now considered an endemic and re-emerging virus throughout Europe. Because of the severe symptoms exhibited by infected animals, BTV infection causes economic losses and impacts the international cattle trade (estimated to be up to US$ 3 billion) (Alkhamis et al. 2020). Although BTV mostly results in clinical symptoms in sheep, cattle act as reservoir hosts and play an important role in BT transmission and epidemiology because they exhibit prolonged viremia (rarely associated with reproductive failure and low milk production) (Alkhamis et al. 2020).

Surveillance for this virus is based on the identification of the pathogen in vectors and symptomatic hosts, as well as the detection of specific antibodies. Bluetongue is endemic among ruminant populations in Italy, and repeated outbreaks are described, especially in small ruminants, while information on circulation in other species is more lacking (Carvelli et al. 2019). The aim of this study was to investigate the BTV seroprevalence in the Campania region, southern Italy.

## Materials and methods

### Sampling and study area

The current study was conducted in Campania (410000000 N-143000000 E), a southern Italian region overlooking the Mediterranean Sea. Due to the promiscuity of ruminants and meteorological conditions, this area favors the circulation of arboviruses. Samples were selected from those collected in a previous study (approved by the Institutional Ethics Committee of the Department of Veterinary Medicine and Animal Production, Centro Servizi Veterinari, Federico II University of Naples, PG/2022/0093419, 20 July 2022) (Ferrara et al. 2023a, b). 218 buffaloes and 250 cattle were sampled. Animals came from 31 separate districts and four provinces (21 cattle herds and 20 water buffalo herds), none of which practiced BTV immunization. Samples were collected at the end of the 2022 vector season (October–November 2022). Data on potential risk variables (species, age, and housing) were collected. Environmental data (annual average temperature, altitude, and distance from the coast) were calculated using the farm's geographical coordinates and data obtained by the Italian National Meteorological Service from meteorological stations located near the sampling farms (http://centrofunzionale.regione.campania.it/#/pages/dashboard).

### Serological analysis

Sera were separated from blood samples by centrifugation at 1000 g for 10 min and kept at -20 °C until testing. A commercial assay (ID Screen® Bluetongue Competition, ID Vet) was used to detect BTV antibodies in each sample. This competitive assay detects antibodies against all BTV serotypes (using the VP7 core protein as antigen) and is selective (does not cross-react with antibodies against the closely related epizootic hemorrhagic disease virus). The assay was performed following the manufacturer's instructions and optical density values at 450 nm were determined using a spectrophotometer. Results were calculated for each serum as a ratio to the mean of negative controls (Sample/Negative control ratios, or S/N) and compared to cut-off values (S/N < 40% are defined as positives).

### Statistical analysis

Prevalence and information on sampled animals were employed for risk factor analysis. The chi-square test was used to carry out a bivariate analysis of the putative risk factors for BTV seropositivity at the animal level. The serological result (positive or negative) was regarded as dependent variable, whereas the information gathered through the epidemiological questionnaires was considered as independent variables. Species, age, province, type of housing, altitude, mean annual temperature, total annual rainfall, and distance from the coast were the independent variables. The correlation between dependent and independent variables was investigated using Chi-square statistics. A p-value of less than 0.05 was considered significant, and a threshold of 0.2 was utilized to select the variables to be included in the multivariate logistic regression (using the forward elimination strategy). Odds ratios (OR) and 95% confidence intervals were calculated to analyze the degree of correlation between independent factors and BTV seropositivity. Fit models were assessed using the Akaike Information Criterion (AIC), and those that best fit the data were chosen. The Variance inflation factor (VIF) was used to test for collinearity. For statistical analysis, MedCalc Statistical Software version 16.4.3 (MedCalc Software, Ostend, Belgium; www.medcalc.org) and JMP version 14.1.0 (SAS Institute Inc.) were used.

## Results

A competitive ELISA was used to screen 468 ruminants for the presence of BTV antibodies and 204 animals tested positive. A prevalence of 43.6% was therefore reported at the individual level, but it was significantly greater at the herd level (85.4%), given that only 6 out of 41 farms did not have any positive animals. No substantial differences were detected between species at the herd or individual level. In fact, seroprevalence varied between 45.2% and 90.5% in cattle and between 41.7% and 80% in buffalo at the individual and herd levels, respectively. The province of Benevento had fewer positive animals than any other province (with a prevalence of approximately 20% compared to a prevalence ranging from 40 to 52% in the other provinces). There were no relationships between high seropositivity and the type of housing evaluated (although grazing animals had greater percentages of positives). Furthermore, animals that were exposed to SBV were no more likely to be positive for BTV (only 21.8% of animals seropositive for SBV testing were also exposed to BTV). Age was significantly associated with a higher probability of infection among the individual risk variables (Table 1). Among the environmental parameters included (average annual temperature, altitude, yearly rainfall, and distance from the coast), the mean annual temperature plays the most important role and is the only factor associated with the greatest seroprevalences (Table 1). Multivariate logistic regression included all variables with a p-value of 0.2 or lower. All variables were included because they satisfied the multicollinearity requirement (VIF less than 2.5). Mean annual temperature was confirmed as the primary risk factor by multivariate analysis. Age was also identified as a risk factor for BTV positivity in multivariate analysis. Adult animals were 1.48 times more likely to test positive for BTV antibodies than younger animals (Table 2). Furthermore, animals raised in regions with an average annual temperature above 16 °C were 1.64 times more likely to test positive (Table 2).

**Table 1:** Univariate investigation (chi-square) assessed individual and environmental possible risk variables for bluetongue virus seropositivity (location, species, housing, outcome to SBV ELISA, annual mean temperature, altitude, yearly rainfall, distance from the coast)

Factor	*n*	Positive	%	95%CI	χ^2^	*p*-value
Total	468	204	43.6	39.1 – 48.08		
Species						
Cattle	250	113	45.2	39.03 – 51.37		
					0.56	0.45
Water buffalo	218	91	41.7	35.2 – 48.29		
Age						
Adult	389	179	46	41.06 – 50.97		
					5.51	0.019
Young	79	25	31.7	21–39 – 41.90		
Location						
Avellino	63	31	49.2	36.86 – 61.55		
Benevento	54	11	20.4	9.63 – 31.11	17.81	< 0.001
Caserta	168	68	40.5	33.05 – 47.90		
Salerno	183	94	51.4	44.12 – 58.61		
Housing						
Fully stallfed	422	178	42.2	37.47 – 46.89		
					3.47	0.063
Partly grazed	46	26	56.5	42.2 – 70.85		
Coexposure with SBV						
Yes	236	102	43.2	36.9 – 49.54		
					0.03	0.871
No	232	102	44	37.58 – 50.35		
Mean annual t°						
≤ 16 °C	221	83	37.6	31.2 – 43.94		
					6.2	0.013
> 16 °C	247	121	49	42.75 – 55.22		
Altitude (a.s.l.)						
≤ 250 m	331	138	41.7	36.38 – 47.00		
					1.65	0.198
> 250 m	137	66	48.2	39.81 – 56.54		
Annual rainwater (cm)						
≤ 1000	124	63	50.8	42.01 – 59.61		
					3.57	0.059
> 1000	344	141	41	35.79 – 46.19		
Distance from the coast						
≤ 20 km	183	73	39.9	32.8 – 46.9		
					1.67	0.196
> 20 km	285	131	46	40.18 – 51.75

**Table 2:** A logistic regression model was used to examine the relationship between putative risk variables (*p* < 0.2), and bluetongue virus seropositivity

Factor	Coefficient (β)	Standard error	OR	OR CI%	*p*-value
Mean annual temperature (> 16 °C)	0.492	0.19	1.64	1.13–2.4	0.01
Age (> 24 months)	0.644	0.265	1.48	1.12–1.69	0.015

## Discussion

The results of this study confirm the trend observed in tropical and temperate regions where BTV has become endemic. In fact, a high prevalence has been observed in countries where the host vector cycle is conserved (as in Italy), not only due to its wide distribution but also as a result of a long-lasting antibody response. When ruminants are infected with BTV, they produce antibodies against both structural (on which the assay used in this study is based) and nonstructural viral proteins (Zhugunissov et al. 2015). BTV-specific neutralizing antibodies are detectable 5–6 days after the infection and can persist for several months, providing long-term resistance to reinfection with the homologous BTV serotype (representing the basis for vaccination) (Ramakrishnan et al. 2006; Maclachlan et al. 2014). Since our sampling coincided with the end of the vector season, we ensured to identify the largest number of positive animals.

The prevalence we observed is in line with that found in other countries where BT is endemic. Great part of the studies were conducted on cattle, describing exposure rates ranging from 27 to 67% in African and Asian countries (Hwang et al. 2019; Bakhshesh et al. 2020). All these studies reported age as a significant risk factor since adult animals are not protected by colostral immunity and interact with new vector seasons during their lives.

However, there is limited information on the diffusion of BT in buffalo in Europe. In fact, most of the studies in this sense are conducted on other continents, finding prevalence very similar to that observed in other ruminants, such as in Asia (Sohail et al. 2019; Rupner et al. 2020). All these data support the central role of buffalo in the epidemiology of this infection, even if there is no data on the duration of the viraemia. The differences observed in these studies could be due not only to a different epidemiological situation but also to the approach used and the timing of sampling (environmental factors can influence the vector season and thus the spread of these infections).

Numerous descriptive studies in the literature provide data about co-infections and co-exposures with BTV and SBV or other arboviruses. In regions where both infections are endemic, the prevalence of SBV is usually higher than that of BTV and the co-exposure rate is rather low (this is described in both domestic and wild animals in several countries). SBV has been described as the predominant viral exposure in captive fallow deer (Wernike et al. 2022). This trend is also confirmed in the roe deer in Flanders (27.9% for SBV and 0% for BTV), in Poland in the wild European bison (76.1% for SBV and 24.7% for BTV), and in Ireland in the red deer (1.5% for SBV and 0% for BTV) (Tavernier et al. 2015; Graham et al. 2017; Krzysiak et al. 2017).

Co-infection with BTV-SBV, as well as with other arboviruses, has been documented in domestic ruminants in Israel (Behar et al. 2022). In contrast, the only European study that has described the degree of co-exposure to SBV and BTV in domestic animals is a 6-year study conducted in Poland, in which, among other factors, exposure to BTV in cattle was a risk factor for SBV positivity (Kęsik-Maliszewska et al. 2021). The lack of association between BTV and SBV exposure and the low rate of co-exposure are highly interesting, especially considering that these viruses share the same vector. Even more surprising, there is no evidence in the literature at the molecular level, and co-infection has not been described in either *Culicoides* or animals. In the absence of scientific confirmation, plausible explanations for these results involving the host or the vector can be proposed. For example, we can consider the degree of competition between the two viruses to infect the vector.

Regarding environmental risk factors, as seen in previous studies carried out on other arboviruses, average temperature is the most impactful factor in determining the effective spread of this infection. It is not surprising that other environmental factors do not affect the prevalence of BTV, as *Culicoides* are now described in Italy at any altitude and in any ecosystem. The success of these infections must be sought in the stratification and in the dispersal of the vector, which can be transmitted over hundreds of kilometers (Mignotte et al. 2021; Karthikeyan et al. 2022).

Certainly, the fact that these arbovirus infections are so common, with even modest rates of co-exposure, should be a reason for significant worry, considering that many nations have extremely strict rules regarding the purchase and sale of genetic material from other countries. Embryos, sperm, and oocytes from seropositive animals cannot be distributed in several European countries. We should thus consider the true damage that these diseases bring to the overall cattle sector rather than focusing on the minor harm they cause to symptomatic animals. Surveillance of this disease is critical for understanding the level of spread in a territory, taking control measures, and therefore accomplishing prevention. In Italy, and in particular in the Campania region, evidence of arbovirus disease in different populations has been described (Montagnaro et al. 2019; Petruccelli et al. 2020) and probably more will be described in the future. Cattle and buffaloes play a fundamental role in the maintenance of some arboviruses, and at the same time, they are important sentinels to prevent any outbreaks. Future investigations are necessary in order to understand the main serotypes circulating in the area as well as the characteristics of the infection in buffalo.

## Conclusions

Bluetongue is widespread among buffalo and cattle herds in the Campania region. Age and mean annual temperature were the most significant risk variables in both univariate and multivariate analyses. BTV prevalence is not affected by SBV seropositivity. Cattle serve as key sentinels to anticipate outbreaks in sheep and goats, in addition to being the major reservoir of infection due to extensive viremia. Further studies are needed to evaluate the duration of viraemia in buffalo and to establish the precise epidemiological role of this species.

## Data Availability

Data sharing not applicable to this article as no datasets were generated or analysed during the current study.
